# Gendered pathways to socioemotional competencies in very young children

**DOI:** 10.1038/s41598-024-56854-0

**Published:** 2024-03-16

**Authors:** Qin Liu, Jing Huang, Melissa Pearl Caldwell, Sum Kwing Cheung, Him Cheung, Tik Sze Carrey Siu

**Affiliations:** 1 The Education University of Hong Kong, 10 Lo Ping Road, Tai Po, New Territories Hong Kong; 2 Lingnan University, New Territories, Hong Kong

**Keywords:** Psychology, Human behaviour

## Abstract

Parent–child and teacher–child relationship closeness have been shown to be crucial for children’s development of socioemotional competencies from preschool to school-age stages. However, less is known about the importance of developing close relationships with young infants and toddlers attending childcare group settings for their early socioemotional development. The current study aimed to address this gap and to explore how child gender may influence the associations. Participants included 378 Hong Kong Chinese children (196 girls; *M*_*age*_ = 22.05 months, *SD* = 9.81 months) enrolled in childcare centres, along with their parents and teachers. Parents reported on children’s socioemotional competencies as well as their relationship closeness with children; teachers reported on their relationship closeness with children. Multiple group structural equation modelling was used to analyse the results. The findings showed that both parent–child and teacher–child closeness were positively associated with children’s social competence, while teacher–child closeness was negatively associated with children’s anxiety behaviour. Parents of girls reported greater parent–child closeness, higher levels of social competence, and higher levels of anxiety behaviours compared to parents of boys. Furthermore, teacher–child closeness was significantly associated with social competence exclusively among girls, while parent–child closeness was significantly associated with anxiety behaviours solely among boys. Findings are discussed in terms of the role of child gender in influencing the associations between parent–child closeness, teacher–child closeness, and children’s socioemotional competencies in the earliest years.

## Introduction

Socioemotional competencies refer to the ability to regulate one’s executive function and behaviour in a social context, including independence and cooperation, affective expression and emotion regulation abilities, and relationship formation abilities during interactions with others such as parents and teachers^1,2^. These competencies are critical to children’s school entry and academic performance^1,3,4^, later social competence and success^5^, and are important predictors of children’s mental health and personal well-being later in life^6–8^. What are some socio-environmental factors contributing to development of socioemotional competencies during childhood? The extant literature has revealed the pivotal roles of parent–child and teacher–child closeness in supporting children’s development of socioemotional competencies^9–16^. Closeness in these relationships is often characterised by warmth, affection, sensitivity, and open communication^17^, and has been identified as a significant and more reliable predictor of relationship quality compared to conflict^9,12,14,17,18^.

However, it should be noted that previous research has primarily examined the associations between parent–child closeness or teacher–child closeness and children’s socioemotional competencies during the preschool and primary education stages (e.g.,^9,12–16^). To date, less empirical attention has been given to investigating whether or how close relationships with significant others (i.e., parents and teachers) may influence the development of socioemotional competencies in infants and toddlers attending childcare group settings^10^. Very young children from 6 months onwards demonstrate a marked onset of socioemotional characteristics, including anxiety, aggression, and social competence^19–22^. Prior research has indicated that after the sixth month young infants undergo a significant shift in their attitude towards human interaction, exhibiting distinct signs of anxiety that frequently manifest as drastic flight responses upon encountering unfamiliar adults^19,20^. Some infants as young as 6 months of age already display early indications of elevated levels of aggression^22^. Furthermore, it has been demonstrated that the socioemotional competencies of young children can be effectively assessed as early as 6 months through parents’ reports and observations^19–22^. Since the beginnings of socioemotional characteristics could be observed from 6 months onwards, we sought to examine the potential antecedents of socioemotional competencies of very young children as the first years represent a critical period for rapid development in socioemotional domains through interactions with important caregivers^6,10^.

There has been burgeoning literature reporting differences in parent–child closeness, in teacher–child closeness, and in children’s socioemotional competencies between boys and girls in childhood and adolescence (e.g.,^15,20,23–26^). Fewer studies, however, have examined these gender differences in children aged under 3 years attending group settings. Moreover, to date, no studies have specifically examined the role of child gender in influencing the associations between parent–child closeness, teacher–child closeness, and children’s socioemotional competencies among infants and toddlers who develop relationships with their significant caregivers. To fill the knowledge vacuum, the current research into infants and toddlers attending group-based childcare settings serves a dual purpose: it examines the associations between parent–child closeness, teacher–child closeness, and children’s socioemotional competencies among very young children enrolled in childcare settings and investigates the potential role of child gender in moderating these associations.

### Theoretical underpinning for the study

Bronfenbrenner’s Process-Person-Context-Time (PPCT) and bioecological model^27,28^, coupled with Bowlby’s attachment theory^29^, guided this study in addressing our research aims. Bronfenbrenner’s Process-Person-Context-Time (PPCT) model and bioecological system perspective foreground the study of child development within four major components (Process, Person, Context, and Time) and consider children’s emotional socialisation as an interwoven and reciprocal process^27,28^. Proximal processes serve as the primary driving force for the development of individuals, characterised by the interactions between the persons (e.g., children and their parents and teachers) or objects in the immediate environment of the child. In addition, characteristics of the child, such as child gender, not only impact their developmental processes but also directly shape their proximal processes^27^.

Importantly, the Context componment of Bronfenbrenner’s bioecological system^28^ emphasises that a child’s unique developmental trajectory is shaped by his or her interactions within various contextual systems (e.g., social and physical environments)^10^. The microsystem, predominantly comprising a child’s social interactions within familial or educational settings, is widely recognised as the contextual system closest to the child^28^. Within this microsystem, the child’s relationship quality with parents in the family and the child’s relationship quality with teachers in education and care group settings play pivotal roles in both positive and negative socioemotional developmental outcomes in children (e.g.,^11,30,31^). While the macrosystem is the outermost layer of influence on the child, its impact extends throughout all other layers (e.g., microsystem)^28^. For instance, child gender can influence the relationship closeness between children and their parents as well as their teachers^9,15,17,32^. One explanation is that child gender could be a manifestation of the macrosystem (e.g., cultural values, beliefs, and expectations) being enacted within the microsystem processes (i.e., family and school education)^33^. Prior research has shown that when parents adhere to traditional cultural values and hold rigid beliefs about gender roles, they are more likely to exhibit gender-differentiated interactions and behaviours that reinforce child gender-role consistent behaviour, such as reinforcing aggressive behaviours or responding punitively to emotional outbursts in their sons but not in their daughters^33,34^.

Children’s attachment refers to the enduring emotional bond established by children with their attachment figures^35^. According to Bowlby’s attachment theory^29^, secure attachment is characterised by the child feeling safe in their relationships with their attachment figures. For children attending childcare centres, secure attachment is established when parents and early childhood teachers consistently provide accessible and sensitive responses to children’s needs^35,36^. As such, children who establish secure attachment with their parents and teachers are more likely to have warm and intimate parent–child and teacher–child relationships compared to those who have insecure attachment with parents and teachers^37,38^. Furthermore, prior studies have indicated that child gender can affect their relationships with both parents and teachers^9,15,17,39^. It is thus important to consider and probe into gender differences in these key relationships. Attachment representations (e.g., warm and intimate parent–child and teacher–child relationships) formed in the earliest years are crucial to child development because these representations shape children’s interpersonal dynamics throughout the lifespan, significantly influencing the unfolding of children’s socioemotional competencies^40,41^.

### Parent–child closeness and children’s socioemotional competencies

Family is the immediate setting children inhabit and is thus the most common microsystem for very young children wherein proximal processes occur^27,28^. This study focused on different-sexed married couples^42^ with an infant or toddler in childcare.

Considerable evidence has indicated the significant impact of close, warm, and intimate parent–child relationships on improving children’s social skills, physical health, mental health, self-regulation abilities, and in reducing children’s problem behaviours^9,10,17,30,40,43^. And there has been no lack of studies examining the asociations between parent–child closeness and children’s socioemotional competencies in preschool settings, but the findings so far have been mixed. For example, Xu et al.^9^ demonstrated a positive association between mother–child closeness and the social skills of preschoolers in China. In contrast, another study conducted by Saral and Acar^16^ in Turkey found that parent–child closeness was not a significant predictor of children’s social competence in preschool years. It should be noted that the associations between parent–child closeness and children’s socioemotional development in infants and toddlers attending childcare group settings in Eastern cultural contexts remain under-studied. Only one U.S. study by Lang et al.^10^ examined relationships between parents and their children aged 1–3 and found that closer parent–child relationships were associated with higher levels of children’s social-emotional competence as indexed by child’s compliance, ability to attend, empathy, and prosocial peer behaviours.

### Teacher–child closeness and children’s socioemotional competencies

High-quality teacher–child relationships established in education and care group settings provide a meaningful context for nurturing children’s socioemotional competencies^11,12,31,36^. García-Rodríguez et al. conducted a systematic review of 24 studies over the last ten years, focusing on children aged 6–13 and their teachers, and revealed significant associations between teacher–child relationship quality and child outcomes such as school liking, peer acceptance, self-concept, emotional regulation, and externalising and internalising behaviour^11^. Additionally, children who had low levels of closeness with their teachers showed higher levels of externalising and internalising behaviour problems in primary schools^44^.

Other studies have also provided evidence that teacher–child closeness serves a protective function for children to develop their socioemotional competencies and behaviours^12–14,45,46^. For example, higher levels of teacher–child closeness were associated with better social, emotional and behavioural classroom adjustment among children receiving special primary education^12^. Cadima et al.^13^ also found that closer teacher–child relationships improved social self-regulation among preschool children who were economically disadvantaged. Moreover, Acar et al. suggested that for primary school children with high levels of shyness, an increase in teacher–child closeness was associated with improved social competence ratings^14^. Note that most previous studies have only examined the relationship between teacher–child closeness and children’s socioemotional competencies among children attending preschools and primary schools (e.g.,^11,12,14,18,47^). To date, few studies have probed into these associations in infants and toddlers who are attending childcare group settings. Given the pivotal role of teachers as significant caregivers in these settings^31^, further research is warranted to complete our understanding of how teacher–child closeness may influence children’s development of socioemotional competencies throughout their childhood.

### The role of child gender in parent–child and teacher–child closeness and in socioemotional competencies

Prior work conducted in school settings has indicated that parents and teachers interact with boys and girls differently. They also play significant roles in providing children with gender socialisation experiences^9,15,17,32,33,48^. These studies have also suggested that parents and teachers, as significant caregivers of very young children, may contribute to shaping gender-typed differences between boys and girls in the earliest years^49^. Numerous studies have shown that parents and teachers tend to have closer relationships with girls compared to boys in early childhood (e.g.,^9,15,17,32^). In a study of 1366 Chinese preschoolers and their parents, Xu et al.^9^ found that parent-daughter dyads had higher levels of closeness than parent-son dyads. This finding is consistent with a U.S. study conducted by Driscoll and Pianta^17^, which showed that fathers reported higher levels of closeness with their daughters than with their sons during preschool to primary school period.

Similar findings have also been observed in teacher–child closeness. Girls are more likely to have a warm and close relationship with teachers than boys in preschools and primary schools^15,32,50^. These observed disparities in teacher–child closeness may be attributed to teachers exhibiting more negative interactions with boys than with girls in school settings. Jones and Dindia conducted a comprehensive meta-analysis of 32 studies within pedagogical settings and suggested that males were subjected to a higher frequency of criticism, reprimands, behavioural warnings, and non-supportive feedback from teachers compared to their female classmates from primary school to college^48^. Nonetheless, up to now, far too little empirical attention has been paid to potential differences in parent–child closeness and in teacher–child closeness between girls and boys in infants-toddlers attending group settings.

Furthermore, prior research has revealed gender differences in children’s socioemotional competencies and behaviours in childhood and adolescence. Boys are proportionately more likely to exhibit externalising problems such as aggression compared to girls during childhood and adolescence^15,16,51^, whereas girls are more likely to experience internalising problems such as anxiety and depression^23–25^. Girls are also more likely to display positive social behaviours, cooperation assertion, prosocial cognitions and behaviour^23,24^. However, gender differences across a wider range of socioemotional competencies among 6-month- to 3-year-old infants and toddlers is currently under-studied. In the context of children at this very young age, prior work seemingly focused on gender differences in one or two specific components of socioemotional competencies (e.g., aggressive behaviours)^26,52,53^. Six months to three years is particularly important for studying gender differences in socioemotional competencies, as this is the period during which many of these gender differences emerge^19–22,53^. Another knowledge vacuum is the potential differences in the associations between parent–child closeness, teacher–child closeness, and children’s socioemotional competencies between boys and girls attending childcare group settings. The current study sought to fill these research gaps.

### Objectives and hypotheses of the study

This study had two objectives. The first objective was to examine the associations between parent–child closeness, teacher–child closeness, and children’s socioemotional competencies (including social competence, anger aggression, and anxiety withdrawal) in very young children aged 6 months to 3 years attending childcare group settings. The second objective was to explore potential differences in the associations among parent–child closeness, teacher–child closeness, and very young children’s socioemotional competencies between boys and girls.

We proposed two hypotheses in this study. First, drawing on Bronfenbrenner’s PPCT model and bioecological system^27,28^, Bowlby’s attachment theory^29^, and existing findings with older children (see^9–11,15,47^), we hypothesised positive associations between parent–child closeness and children’s social competence and between teacher–child closeness and children’s social competence in 6-month- to 3-year-olds attending group settings. Second, building on prior research^9,15,17,32^, we hypothesised that girls will have higher levels of closeness with their parents and teachers in childcare centres compared to boys. Lastly, in an exploratory manner, we investigated possible differences in the associations between parent–child closeness, teacher–child closeness, and children’s socioemotional competencies in very young children between the two gender groups.

## Method

### Participants

We sent invitation emails to 14 standalone childcare centres and 30 kindergarten-cum-child care centres (i.e., those attached to a kindergarten) in Hong Kong to recruit children aged 6 months to 3 years and their parents and teachers. Eight standalone childcare centres and ten kindergarten-cum-child care centres replied and agreed to distribute the invitation letter and information sheet to their early childhood teachers and children’s parents. A total of 378 children (196 girls) and their parents participated. The children’s age ranged from 6.06 months to 43.77 months (*M*_*age*_ = 22.05 months, *SD* = 9.81 months; see Table 1). Note that we provided three options (i.e., *female*, *male*, and *prefer not to say*) for the question on child’s gender. All parents indicated either female or male as their child’s gender. Over half (52.9%) of the participants’ monthly household incomes were in the range of HK$20,001–60,000, while 33.3% of those were HK$60,001 or above. According to the report by the Census and Statistics Department of Hong Kong^54^, the median monthly household income in Hong Kong was HK$30,000. Therefore, our sample mostly came from lower-middle-class and middle-class families in Hong Kong. Questionnaires were employed to gather background information from parents, including their monthly household income and mothers’ education level. More than half of the mothers (56.1%) had at least a Bachelor’s degree qualification. The children’s lead teachers also participated by completing a questionnaire. Among all the participating teachers, 66.9% had less than 15.5 years of teaching experience with children aged 3 and below. Also, 88.1% of the teachers had received professional training for working with children aged 3 and below. The sample size used in this study was sufficient for conducting our data analyses^55^ and was also similar to that used in prior similar research on very young children (e.g.,^10,56,57^).

**Table 1:** Demographic characteristics.

	Children (*N* = 378)		
	*N*	*%*	
Child gender			
Male	182	48.2	
Female	196	51.8	
Maternal education			
Secondary education or below	163	43.1	
Bachelor’s degree or above	212	56.1	
Unavailable	3	0.8	
Monthly household income			
≤ HK$ 20,000	40	10.6	
Between HK$ 20,001 and HK$ 60,000	200	52.9	
> HK$ 60,001	126	33.3	
Unavailable	12	3.2	
	*M*	*SD*	Range
Children’s age (in months)	22.05	9.81	6.06–43.77

### Procedures

Ethical approval for this study was obtained from the Human Research Ethics Committee of the Education University of Hong Kong. All the methods were performed in accordance with the guidelines and regulations from the approving committee.We obtained the contact information of early childhood group settings offering childcare services to children from birth to three from the website of the Social Welfare Department of Hong Kong, which is publicly available. All the participating parents and teachers provided written informed consent to participate in this study. The parents were invited to complete a background questionnaire about demographic information, and to complete questionnaires on their child’s social competence and behaviour and their relationship closeness with their child. The lead teachers were also asked to complete a questionnaire about the relationship closeness with their students.

### Measures

#### Social competence and behaviour

The Social Competence and Behaviour Evaluation Scale—Short Form (SCBE-30)^2^ was used to measure parents’ perspectives of children’s patterns of maladaptive behaviours, emotion regulation and expression, and social competence. The SCBE-30 has demonstrated its validity and reliability for investigating social competencies of very young children^58^, as well as its applicability to studying young Chinese children^50^. The SCBE-30 assesses three dimensions in children: (i) *Anger-Aggression*, (ii) *Anxiety-Withdrawal*, and (iii) *Social Competence*. The scale consists of 30 items, with 10 items for each dimension. The items were rated on a 6-point Likert scale ranging from 1 (‘*never*’) to 6 (‘*always*’). The possible score range for each subscale was 10–60. In this study, the internal reliability coefficients (alpha) of the *Anger-Aggression*, *Anxiety-Withdrawal*, and *Social Competence* subscales were 0.78, 0.77, and 0.78, respectively. A higher score on *Anger-Aggression* indicates the child exhibits more aggressive behaviours; a higher score on *Anxiety-Withdrawal* indicates the child exhibits more anxious behaviours; and a higher score on *Social Competence* indicates the child exhibits more prosocial behaviours.

#### Parent–child closeness

The *Closeness* subscale of the Child-Parent Relationship Scale—Short Form (CPRS-SF)^17^ was used to measure parents’ perception of warmth, affection, and open communication in their relationships with their child^43,57^.We used this measure because a high level of internal consistency of the *Closeness* subscale was found among Chinese parents with their young children in previous research (e.g.,^43,56,57^). Moreover, the CPRS-SF was validated for assessing the relationship quality between parents and very young children below three years of age in prior studies^10,56,59^. The *Closeness* subscale consists of 7 items (e.g., “I share an affectionate, warm relationship with my child”). The items were scored on a 5-point Likert scale ranging from 1 (‘*definitely does not apply*’) to 5 (‘*definitely applies*’). The possible score range for the *Closeness* subscale was 7–35. A higher score on the *Closeness* subscale reflects a warmer and more intimate relationship. The internal reliability coefficient (alpha) of the scale was 0.79.

#### Teacher–child closeness

The *Closeness* subscale of the Student–Teacher Relationship Scale—Short Form (STRS-SF)^60^ was used to measure teachers’ perception of warmth, affection, and open communication in their relationships with the child. Prior studies have consistently demonstrated good internal consistency of the *Closeness* subscale in samples of young Chinese children (e.g.,^46,56,57,61–63^). In this study the internal reliability coefficient (alpha) of the *Closeness* subscale was 0.81. The *Closeness* subscale consists of 7 items, and the items were rated on a 5-point Likert scale ranging from 1 (‘*definitely does not apply*’) to 5 (‘*definitely applies*’). The possible score range for the *Closeness* subscale was 7–35. A higher score on the *Closeness* subscale indicates a warmer and more intimate teacher-student relationship.

### Ethical approval and informed consent

The present research involves human participants. All adult participants (parents and childcare teachers) gave their written informed consent prior to participating in the study. The parents also gave consent on behalf of their child.

## Data analyses and results

### Descriptive statistics and correlations

We conducted preliminary analyses with SPSS 28.0, including descriptive statistics (e.g., means, standard deviations, ranges) and bivariate correlations among the study variables. The descriptive statistics and bivariate correlations are summarised in Table 2. Overall speaking, parent participants reported medium to high levels of closeness with their child (*M* = 27.63, *SD* = 3.72). Teacher participants also reported medium to high levels of closeness with the children (*M* = 26.82, *SD* = 4.08). Parent–child closeness and teacher–child closeness were not mutually correlated (*r* = 0.03). Parent–child closeness was positively correlated with children’s social competence (*r* = 0.31) and negatively correlated with children’s anxiety withdrawal (*r* = − 0.21). Similarly, teacher–child closeness was positively correlated with children’s social competence (*r* = 0.26) and negatively correlated with children’s anxiety withdrawal (*r* = -− 0.22). In addition, we found that children’s age was correlated with both parent–child closeness (*r* = 0.36) and teacher–child closeness (*r* = − 0.12). Therefore we controlled for children’s age in all the subsequent analyses.

**Table 2:** Descriptive statistics and bivariate correlations among study variables.

	1	2	3	4	5	6
1. Age (in months)	–					
2. Parent–child closeness	.36**	–				
3. Teacher–child closeness	− .12*	.03	–			
*Socioemotional competencies*						
4. Social competence	.14	.31**	.26**	–		
5. Anger aggression	.05	− .12	− .04	.10	–	
6. Anxiety withdrawal	.07	− .21**	− .22**	− .08	.47**	–
Minimum	6.06	14.00	14.00	13.00	10.00	12.00
Maximum	43.77	35.00	35.00	49.00	38.00	40.00
Mean	22.05	27.63	26.82	35.71	23.68	24.88
Standard deviation	9.81	3.72	4.08	6.85	5.67	5.69

**p < .01, *p < .05.

### Multiple group structural equation modelling

The present data had a hierarchical structure for the 378 participants came from 18 group settings (classrooms). Hence, multilevel modelling was presumably preferred to analyse the nested data. Nonetheless, we tested unconditional multilevel models and calculated ICCs to estimate the proportion of outcome variation at the classroom level. Results indicated that the ICCs for social competence, anxiety-withdrawal, and anger-aggression (key outcome variables) were 0.042, 0.015, and 0.024, respectively, which indicate small within-school dependence. Previous research has suggested that ICCs should be greater than 0.05 to ensure sufficient between-cluster variability to support multilevel analysis^64^. On the basis of this recommendation, we used single-level models in all subsequent analyses.

#### Measurement invariance testing

**Figure 1:**
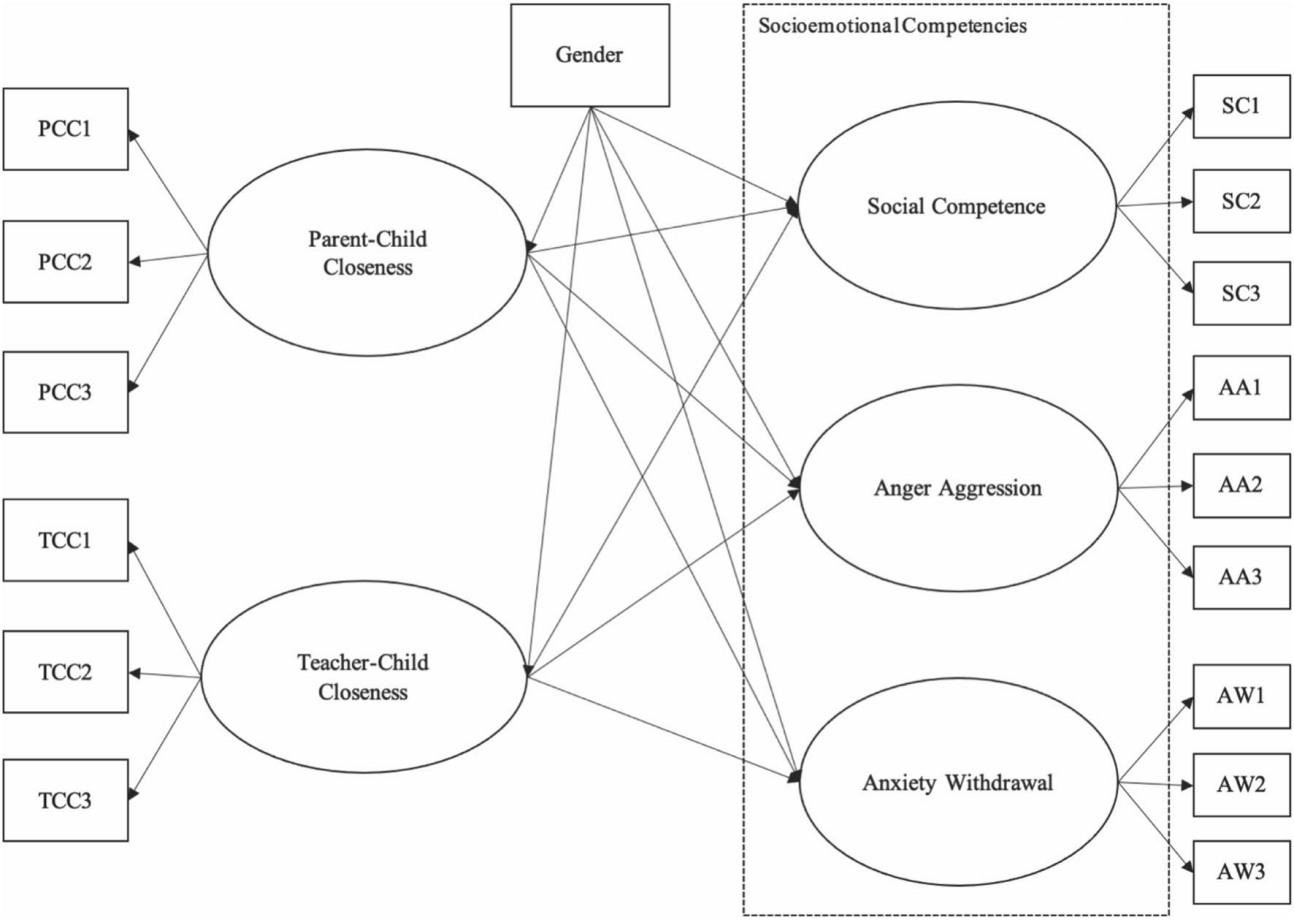
Hypothesised model.

As one purpose of the present study was to examine gender differences in the hypothesised model (Fig. 1) using a multiple group SEM model, measurement invariance of the scales measuring the study variables needs to be established. Measurement invariance was used to ensure that observed gender differences in the hypothesised model were not influenced by measurement biases and that the scales being compared were measured consistently between the gender groups in this study. Although models with item-level data as indicator variables generally show poorer fit than those using item parcels as indicator variables, item-level data should still be used for measurement invariance tests because using item parcels can potentially mask a lack of invariance^65^. No outliers were found prior to the analysis. Table 3 shows the results of measurement invariance testing for all the scales between the gender groups. The results showed that, for the parent–child closeness scale, the changes in χ^2^ for configural and metric invariance models were significant (Δχ^2^ (6) = 22.58, *p* = 0.001) and the changes in χ^2^ for metric and scalar invariance models were non-significant (Δχ^2^ (6) = 3.56, *p* = 0.737). Furthermore, the alternative fit index (i.e., ΔCFI = –0.009 and 0.003, respectively) suggested that the factor loadings and latent means were invariant between the gender groups. Likewise, the scalar measurement invariance between the gender groups was established for the scales measuring teacher–child closeness, social competence, anger aggression, and anxiety withdrawal.

**Table 3:** Measurement invariance for all scales between the gender groups.

Measurement invariance model	Model fit			Comparison of models		
	*χ*^*2*^	*df*	CFI	Δ*χ*^*2*^	Δ*df*	ΔCFI
Parent–child closeness				Metric versus Configural		
Configural	189.51***	28	0.765	22.58**	6	− 0.009
Metric	212.09***	34	0.756	Scalar versus Metric		
Scalar	215.64***	40	0.759	3.56	6	0.003
Teacher–child closeness				Metric versus Configural		
Configural	242.55***	28	0.741	7.14	6	− 0.001
Metric	249.69***	34	0.740	Scalar versus Metric		
Scalar	252.22***	40	0.745	1.53	6	0.005
Social competence				Metric versus Configural		
Configural	127.83***	70	0.867	4.23	9	0.009
Metric	132.06**	79	0.876	Scalar versus Metric		
Scalar	140.03**	88	0.979	7.98	9	0.003
Anger aggression				Metric versus Configural		
Configural	172.33***	70	0.789	20.33*	9	− 0.008
Metric	192.66***	79	0.781	Scalar vs. Metric		
Scalar	206.82***	88	0.772	14.15	9	− 0.009
Anxiety withdrawal				Metric versus Configural		
Configural	205.37***	70	0.789	44.33***	9	− 0.008
Metric	249.70***	79	0.781	Scalar versus Metric		
Scalar	259.69***	88	0.780	9.99	9	− 0.001

*χ*^*2*^ = chi square test, *df* = degrees of freedom, CFI = comparative fit index.

*p < .05, **p < .01, ***p < .001.

#### Parent–child closeness, teacher–child closeness, and children’s socioemotional competencies

The first objective of this study was to investigate the associations between parent–child closeness, teacher–child closeness, and children’s socioemotional competencies (i.e., social competence, anger aggression, and anxiety withdrawal). The hypothesised model (Model 1; see Fig. 1) yielded an excellent fit to the data (*χ*^2^ (101) = 201.55, *p* = 0.000, CFI = 0.940, TLI = 0.919, RMSEA = 0.053, 90% confidence interval [0.042, 0.063]). Table 4 presents the standardised coefficients for the hypothesised SEM model (Model 1) and multiple group SEM model (Model 2). The results showed that both parent–child closeness (*β* = 0.390, *p* = 0.000) and teacher–child closeness (*β* = 0.153, *p* = 0.001) were positively associated with children’s social competence. Furthermore, the results of the Wald chi-square test demonstrated a significant difference between the effect of parent–child closeness on children’s social competence and the effect of teacher–child closeness on children’s social competence (*χ*^2^ (1) = 6.79, *p* = 0.009). Specifically, parent–child closeness had a stronger effect on children’s social competence than teacher–child closeness did. However, the results showed non-significant relationships between parent–child closeness and children’s anger aggression (*β* =  − 0.043, *p* = 0.642), and between teacher–child closeness and children’s anger aggression (*β* =  − 0.056, *p* = 0.478). In addition, there was a negative association between teacher–child closeness and children’s anxiety withdrawal (*β* =  − 0.146, *p* = 0.032), whereas there was a non-significant relationship between parent–child closeness and children’s anxiety withdrawal (*β* =  − 0.137, *p* = 0.068).

**Table 4:** Standardised coefficient for the hypothesised model.

	Whole sample	Male	Female
PCC → SC	0.390 (0.052)***	0.582 (0.094)***	0.483 (0.089)***
TCC → SC	0.153 (0.047)**	0.115 (0.099)	0.417 (0.083)***
PCC → AA	− 0.043 (0.092)	− –0.178 (0.136)	− 0.091 (0.128)
TCC → AA	− 0.056 (0.079)	− 0.156 (0.095)	0.091 (0.110)
PCC → AW	− 0.137 (0.075)	− 0.244 (0.108)*	− 0.103 (0.110)
TCC → AW	− 0.146 (0.068)*	− 0.156 (0.095)	− 0.101 (0.102)
Age → PCC	0.076 (0.052)	0.066 (0.085)	0.375 (0.068)***
Age → TCC	− 0.018 (0.057)	− 0.008 (0.080)	− 0.083 (0.080)
Age → SC	− 0.025 (0.034)	− 0.093 (0.073)	0.268 (0.094)***
Age → AA	− 0.085 (0.059)	− 0.165 (0.090)	0.056 (0.130)
Age → AW	− 0.114 (0.057)	− 0.175 (0.080)*	0.160 (0.101)
Gender → PCC	0.330 (0.054)***		
Gender → TCC	0.029 (0.058)		
Gender → SC	0.587 (0.042)***		
Gender → AA	0.161 (0.087)		
Gender → AW	0.141 (0.065)*		

*AA* anger aggression, *AW* anxiety withdrawal, *PCC* parent–child closeness, *SC* social competence, *TCC* teacher–child closeness.

*p < .05, **p < .01, ***p < .001.

#### Gender differences

The second objective of this study was to explore possible differences in parent–child closeness, teacher–child closeness, and children’s socioemotional competencies (i.e., social competence, anger aggression, and anxiety withdrawal) between the gender groups. We also examined whether gender differences existed in the hypothesised associations between the study variables. First, gender was included in the hypothesised model (Model 1) to examine gender differences in parent–child closeness, teacher–child closeness, and children’s socioemotional competencies. The results shown in Table 4 demonstrated that there was a significant difference in parents’ perceptions of parent–child closeness between boys and girls (*β* = 0.330, *p* = 0.000). Specifically, parents of girls reported greater parent–child closeness than parents of boys. However, no significant gender difference in teacher–child closeness was found (*β* = 0.029, *p* = 0.616). The results also indicated significant gender differences in children’s social competence (*β* = 0.587, *p* = 0.000) and anxiety withdrawal (*β* = 0.141, *p* = 0.029). Specifically, parents rated girls as higher in social competence and anxiety withdrawal than boys did. However, the association between gender and children’s anger aggression was found to be non-significant (*β* = 0.161, *p* = 0.063).

Second, a multiple group SEM model was estimated to explore gender differences in the associations between parent–child closeness, teacher–child closeness, and children’s socioemotional competencies. The multiple group model showed a good fit to the data (*χ*^2^ (202) = 282.15, *p* = 0.000, CFI = 0.947, TLI = 0.937, RMSEA = 0.047, 90% confidence interval [0.033, 0.060]). As shown in Table 4, there was a positive association between parent–child closeness and children’s social competence in both gender groups (*β*_M_ = 0.582, *p* = 0.000; *β*_F_ = 0.483, *p* = 0.000). The Wald chi-square test showed no significant difference in the relationship between parent–child closeness and children’s social competence between boys and girls (*χ*^2^ (1) = 1.427, *p* = 0.232). Furthermore, teacher–child closeness was positively related to children’s social competence for girls (*β*_F_ = 0.417, *p* = 0.000). However, there was no significant association between teacher–child closeness and children’s social competence for boys (*β*_M_ = 0.115, *p* = 0.245). Given that both parent–child closeness and teacher–child closeness were found to be positively associated with children’s social competence for girls, a Wald chi-square test was also run to examine possible difference in the two associations. The results indicated no significant difference (*χ*^2^ (1) = 0.442, *p* = 0.506). In terms of parents’ rating children’s anger aggression, for both boys and girls, the relationship between parent–child closeness and children’s anger aggression was non-significant (*β*_M_ = –0.178, *p* = 0.190; *β*_F_ = − 0.091, *p* = 0.477). Likewise, the results showed no significant association between teacher–child closeness and children’s anger aggression in both gender groups (*β*_M_ = − 0.217, *p* = 0.071; *β*_F_ = 0.091, *p* = 0.408). With regard to children’s anxiety withdrawal, parent–child closeness was found to be negatively related to children’s anxiety withdrawal for boys (*β*_M_ = − 0.244, *p* = 0.024), whereas the link between parent–child closeness and children’s anxiety withdrawal was non-significant for girls (*β*_F_ = − 0.103, *p* = 0.348). In addition, the results showed no significant relationships between teacher–child closeness and children’s anxiety withdrawal for both boys (*β*_M_ = − 0.156, *p* = 0.103) and girls (*β*_F_ = − 0.101, *p* = 0.323).

## Discussion

The present study aimed to contribute new knowledge by examining the associations between parent–child closeness, teacher–child closeness, and children’s socioemotional competencies in 6-month- to 3-year-old infants and toddlers enrolled in education and care group settings, an understudied population in early childhood research. This study also sought to explore how these associations may differ between very young boys and girls. We found that both high levels of parent–child closeness and teacher–child closeness were associated with better social competence in 6-month- to 3-year-olds attending group settings. High levels of teacher–child closeness were associated with less anxiety behaviour in very young children. Notably, we observed gender differences in these associations. Teacher–child closeness was significantly associated with social competence exclusively among girls, while parent–child closeness was significantly associated with anxiety behaviours solely among boys. Moreover, this study revealed that parents of girls rated higher closeness with their children than parents of boys, and parents of girls also rated their children as more socially competent.

### Associations between parent–child closeness, teacher–child closeness and children’s socioemotional competencies

As predicted, our findings indicated that both parent–child closeness and teacher–child closeness were positively associated with children’s social competence in very young children under age 3 attending group settings. Our findings align with prior studies conducted with preschool and primary school children (e.g.,^9,12,15,16,47^), suggesting that these positive associations are not limited to older children at the preschool and primary school stages. These findings are not surprising, as warmer and more intimate relationships with parents and teachers had consistently been shown to support children’s development of social competence in numerous prior studies^11–13,15,30,31,36^. Nevertheless, the present findings are notable for contributing to a more complete understanding of socioemotional development in early years for we gathered new evidence from the youngest age group (i.e., 6-month- to 3-year-olds) in Eastern cultural contexts. The findings lend further credence to Bronfenbrenner’s theoretical framework^27,28^ by demonstrating the pivotal role of very young children’s close relationships with significant figures in the family and educational settings (i.e., the microsystem) in fostering children’s socioemotional competence. That is, the daily activities and interactions occurring in family and childcare centres (i.e., proximal process) serve as important driving forces supporting children to acquire competence in social interactions.

Furthermore, our findings complement existing studies on the associations between teacher–child closeness and anxiety behaviours during childhood (e.g.,^36,46^). O’Connor et al. conducted longitudinal studies within Western cultural contexts to examine these associations during early and late childhood and found that low levels of teacher–child closeness were associated with high levels of internalising behaviours in children^36^. These findings were consistent with findings from Eastern cultural contexts^46^, indicating that teacher–child closeness was negatively related to internalising problems in preschool children. Additionally, O’Connor et al. demonstrated that teacher–child closeness remained a significant predictor of children’s internalising behaviours during the fifth-grade year. Our results extend the prior findings^36,46^, revealing that high levels of teacher–child closeness were associated with less anxiety behaviours in children as young as 6 months to 3 years of age attending group settings.

Prior evidence has indicated that teacher–child closeness may influence the development of children’s externalising behaviours in early childhood^36^. It is noteworthy that neither parent–child closeness nor teacher–child closeness was associated with children’s aggressive behaviour in our sample of 6-month- to 3-year-olds attending group settings. Nevertheless, our findings are partially consistent with Lang et al.^10^, a study that similarly involved very young children but from Western cultural contexts. Specifically, Lang et al.^10^ found that parent–child closeness was not significantly associated with the levels of children’s externalising problems as indexed by the child’s level of impulsivity, aggression, defiance, and aggression toward peers. While we acknowledge that more evidence is needed to substantiate our null results found in very young children below age 3, we speculate that the lack of associations between parent–child and teacher–child closeness and children’s aggressive behaviour might be aligned with previous observations that relational conflicts rather than closeness contribute to children’s negative behavaiour^56,63^.

One interesting observation from the present study was the positive association between child age and the levels of parent-rated parent–child closeness, while a negative correlation was found between child age and the levels of teacher-rated teacher–child closeness. Although these associations were not statistically significant, they provide new insights into the relationship between child age, parent–child closeness, and teacher–child closeness for very young children attending centre-based settings. To explain the positive association between child age and parent–child closeness, we speculate that parents tend to know their children better over time. This increasing knowledge about their child, in turn, enhances parents’ confidence in addressing their child’s needs, fostering positive interactions, and ultimately bolstering bonding and closeness between the parent–child dyad^10,17^. In contrast, the structure of centre-based childcare, which often does not follow a continuity-of-care model and thus a lack of continuing teacher–child relationships across the two to three years spent in the centre, may not be favourable to nurturing teacher–child closeness across the years. Other structural characteristics of childcare centres, such as large group sizes or high child-teacher ratios^66^, may also influence the levels of teacher–child closeness perceived by teachers. Moreover, teachers in childcare settings who lack sufficient support, such as in-service professional training^66^, may encounter challenges in establishing a pattern of closeness similar to that of parents as children age.

### Gender differences in parent–child and teacher–child closeness and in children’s socioemotional competencies

Consistent with our hypothesis, the present findings showed that parents of girls rated higher closeness with their children than parents of boys in our group of very young children attending group settings. Our findings align with prior research examining gender differences in parent–child closeness during preschool and primary school periods^9,17^ and contribute additional evidence from the youngest age group. One explanation for these findings is that parents may exhibit different behaviour in socialising boys’ and girls’ emotional competencies, affected by gender stereotypes^67,68^. For exmaple, parents may show greater emotional engagement and discuss emotions more frequently with girls than with boys. The differential interaction patterns with boys and girls may contribute to different levels of parent–child closeness between the gender groups^33,49^. Another possible explanation is that parent-rated parent–child closeness was also influenced by gender stereotypes^68^. Specifically, in this study, parent–child closeness was measured based on parents’ reports rather than direct observation. As such, parents’ perceptions of closeness with their child could be influenced by gender stereotypes (i.e., parents’ feeling closer to girls than boys) rather than reflecting actual levels of parent–child closeness.

In contrast, we found no significant differences in teacher–child closeness between very young boys and girls attending group settings. This contrasts with a prior meta-analytic study reporting that boys experienced more neutral and negative interactions with teachers than girls did from primary school to college^48^. Spilt et al.^32^ also examined trajectories of teacher–child closeness for boys and girls separately across the primary school years and found that boys exhibited lower levels of teacher–child closeness compared to girls. We argue that our findings could be attributed to the special nature of teacher–child relationships in childcare settings compared to those in preschool or primary schools, as the caregiving context in childcare settings is more intimate^31^. For example, teachers in childcare centres spend a substantial amount of time engaging in daily care routines such as feeding, soothing, and changing nappies for both boys and girls^31^. Consequently, compared to those in preschool and school settings, teachers in childcare centres are more likely to have a comparable amount of intimate interactions between boys and girls. Having said that, a large-scale study is warranted to further examine how child gender may influence teacher–child closeness across childcare, preschool, and primary school contexts. The findings could help clarify whether gender differences in teacher–child closeness only emerge when children enter preschool. Indeed, previous research has shown that early years’ educational settings in Eastern cultures are not gender-neutral^39^.

Furthermore, significant differences were identified between boys and girls in terms of parents’ reports on the social competence and anxiety behaviours of children aged 6 months to 3 years attending group settings. No significant gender differences were observed in their aggressive behaviour. Our findings support the proposition that girls tend to demonstrate higher levels of social competence compared to boys within the preschool context^15^. Such findings align with the notion that gender roles in Eastern cultures are closely tied to stereotypes^67^. For instance, in Chinese culture, there exists a prevailing expectation that girls should exhibit qualities such as caring, accommodation, and empathy to a greater extent than boys^67,68^. Such cultural expectations also profoundly influence how parents and teachers socialise children’s emotional competencies, potentially leading to better sociosocial competence in girls. Likewise, cultural values and expectations seem relevant in interpreting the different levels of anxiety behaviour in girls versus boys^25^. Consistent with previous studies involving older children and adolescents^23–25^, our findings reveal that parents reported higher levels of anxiety behaviours among girls compared to boys in our sample of 6-month- to 3-year-olds attending group settings. Our finding is also consistent with the pattern observed across the lifespan, where females experience higher levels of anxiety compared to males^24^. There is growing evidence to suggest that, in addition to biological factors (e.g., genetics) and psychological factors (e.g., lower self-esteem), sociocultural factors (e.g., stereotypical beliefs and expectations) also contribute to the disparities in anxiety disorders observed between females and males^69^. Specifically, the presence of distinct cultural expectations imposed on girls, where behaviours like shyness, anxiety, and fear are more likely to be rewarded and accepted by adults in girls compared to boys, may contribute to elevated levels of anxiety among girls^70^.

However, our study did not find significant differences in parents’ perceptions of aggressive behaviour between very young boys and girls. Although prior evidence from Western cultural contexts has revealed that boys tend to exhibit higher levels of aggression than girls during childhood and adolescence^26,71^, the debate regarding the emergence of such gender difference in children’s aggressive behaviour persists. For instance, Alink et al.^53^ examined the aggression levels of children aged 10–50 months in the Netherlands and found that gender differences in aggressive behaviour may emerge after 24 months of age. However, other studies have found evidence of gender differences in aggressive behaviour as early as 17 months of age^26,52^. Our findings suggest that further research is needed to determine the age at which the gender difference in aggression becomes apparent.

### Gender differences in the associations between parent–child closeness, teacher–child closeness, and children’s socioemotional competencies

A key contribution of this study is to provide new evidence on the role of child gender in influencing the associations between parent–child closeness, teacher–child closeness, and children’s socioemotional competencies in infants and toddlers attending group settings, an understudied age group deserving more empirical attention. We obtained two major findings from the moderation analyses. The first one is that child gender moderates the association between teacher–child closeness and children’s social competence. Specifically, teacher–child closeness is related to social competence in girls but not in boys among our infant–toddler sample. This finding aligns with the results of Mohamed et al.^15^, which showed that girls’ relationship closeness with teachers had a stronger predictive effect on social skills compared to boys in preschools. Therefore, our finding joins previous evidence to corroborate the general notion that close and intimate teacher–child relationship, which is characterised by warmth, affection, respect, open communication, appear to be more advantageous for girls than for boys during early childhood^15^.

It is interesting to note that, as discussed earlier, the present results showed no gender differences in teacher–child closeness in infants and toddlers attending group settings. In other words, though our early childhood teachers reported similar levels of relationship closeness with boys and girls in their childcare centres, our findings suggest that the very young girls’ social competence are more susceptible to their closeness with teachers compared to the very young boys. More research is obviously needed to replicate the finding and to consider why very young girls, compared to their male counterparts, appear to be more influenced by their relationship closeness with teachers in childcare group settings. Moreover, we call for larger scale research investigating how child gender may influence teacher–child closeness and the association between teacher–child closeness and children’s development across childcare, preschool, and primary school settings in the same cultural context. The findings will extend our current results by determining whether development in young boys and girls continue to be differentially influenced by their relationship closeness with teachers, or if different patterns of results emerge as children grow from early to late childhood.

The second moderation finding is that relationship closeness between parents and children was negatively associated with anxiety behaviours in our 6-month- to 3-year-old boys, but not girls. The finding is partially consistent with previous research showing that positive parenting, early secure parent–child attachment, and high levels of parent–child closeness were linked to less internalising behaviours in children, such as inhibition, anxiety, and depression^9,10,72^. However, we did not observe a significant association between parent–child closeness and anxiety behaviours in very young girls in this study. These moderation findings suggest that very young boys, compared to their female counterparts, could benefit from close and intimate relationships with their parents as far as anxiety behaviour is concerned. If we consider this finding in conjunction with the result that very young girls exhibited higher levels of anxiety and withdrawal behaviours compared to very young boys attending group settings, it seems that girls in early childhood already face a heightened risk of developing anxiety problems.

In sum, the present study not only supplies the much-needed empirical data from non-Western education and care contexts but also expands previous research with older children conducted in preschool or primary school settings^15,32,50^. Importantly, our findings suggest that child gender is an important factor to consider when deliberating the relationship between teacher–child closeness and children’s social competence and the relationship between parent–child closeness and children’s anxiety behaviours in infants and toddlers enrolled in group settings.

### Limitations and future research directions

Several limitations should be noted when interpreting the present results. First, though the measures used in this study have been widely used and validated, our data was all reported by parents and teachers. Future research should use additional measurements such as observation and ratings by independent researchers to triangulate the data. Second, the participants of this study were predominantly from lower-middle and middle-class families in Hong Kong, which limits the generalisability of the findings to families from other socioeconomic classes in Hong Kong. Moreover, it remains to be seen whether our findings are unique to the Hong Kong Chinese context or are applicable to other cultures. Further research involving samples from diverse cultural contexts is warranted to scrutinise the replicability of our findings. Third, our data is cross-sectional in nature. Hence, we are unable to make causal inferences about the associations we found. We suggest conducting longidutinal studies with cross-lagged panel designs so that reciprocal relationships or directional influences between the variables could be drawn. Fourth, although we used multiple group SEM analyses to examine child gender differences in our hypothesised model, the number of childcare centres (*N* = 18) in this study was insufficient to support multilevel analyses. Future research should engage a larger number of settings/classrooms to facilitate investigation of various complex patterns. For instance, by considering different combinations of high and low levels of closeness in each setting, future studies could gain insights into the interconnectedness between family and education settings and potential effects of these associations on children’s developing socioemotional competencies.

### Conclusion and implications

The present study expands our knowledge on the roles of parent–child closeness and teacher–child closeness in influencing children’s socioemotional competencies by gathering new evidence from 6-month- to 3-year-old infants and toddlers receiving education and care in group settings. First, we obtained corroborative evidence that very young children who have warmer and more intimate relationships with their parents and teachers tend to exhibit better social competence, a finding similar to prior results with older age groups. Second, child gender was found to be an important factor influencing the associations relationship closeness with parents and teachers and children’s socioemotional capacities. Specifically, teacher–child closeness was positively related to social competence exclusively in very young girls, whereas parent–child closeness was related to anxiety behaviours solely in very young boys.

This study highlights the significance of building close and intimiate relationships among children, parents, and teachers for very young children’s development of socioemotional competence in their earliest years. Bronfenbrenner’s theoretical models suggest that children’s socialisation occurs within reciprocal processes across various contextual systems. Our findings also suggest the potential influences of sociocultural values, beliefs, and expectations (elements of the macrosystem) on parent–child closeness and teacher–child closeness. These influences are enacted within the microsystem and are likely to be manifested through the socialisation of child gender within family and childcare settings. Understanding these dynamics may help us identify actionable targets for parent education and teacher education programmes aimed at supporting children development and early learning. To gain a more nuanced understanding of how parents and teachers may support children to develop socioemoeional competencies, we think that large-scale longitudinal research is warranted to track the development of gender diffiences in these associations from early infancy to late childhood and to explore the long-term effects of these potentially different trajectories on children’s outcomes.

## Data Availability

The datasets generated during the current study are available from the corresponding author upon reasonable request.
